# More extremely hot days, more heat exposure and fewer cooling options for people of color in Connecticut, U.S.

**DOI:** 10.1038/s42949-024-00186-5

**Published:** 2024-11-03

**Authors:** Shijuan Chen, Katie Lund, Colleen Murphy-Dunning, Karen C. Seto

**Affiliations:** 1 Yale School of the Environment, Yale University, New Haven, CT 06511 USA; 2 Hixon Center for Urban Sustainability, Yale University, New Haven, CT 06511 USA

**Keywords:** Environmental impact, Climate change

## Abstract

It is well-documented that people of color in the U.S. are disproportionately exposed to extreme urban heat. However, most studies have focused on large cities for one point in time, and less is known about how heat exposure changes over time in smaller cities. Here, we present a study of the changing nature of urban heat exposure and cooling strategies for ten cities in Connecticut in the U.S. Our results show that people of color experience more heat exposure and fewer adaptation strategies. They experienced higher overall temperatures, more extremely hot days, and larger increases in heat exposure. Also, they have lower air conditioning ownership rates and lower tree cover. Taken together, the results indicate that people of color are not only exposed to higher temperatures but also disproportionately exposed to increasing temperatures over time. With lower heat adaptation capacity, people of color are more vulnerable to increasing urban heat.

## Introduction

Urban heat exposure will increase due to global warming and changes in urban areas, including urban expansion and the growth of urban population^1,2^. High heat exposure can lead to negative health, economic, and environmental consequences^3,4^. In many places where average temperatures are already high, temperature increases of 1.5° to 2 °C would significantly increase heat mortality^3,5^. In the United States (U.S.), heat exposure is the largest cause of weather-related fatalities^6^. Heat exposure can also cause non-fatal illness, such as heat stroke, fainting, and heat rash^7,8^, as well as negative economic consequences, including increasing healthcare costs^9^, heat-induced reduction in worker productivity^10^, and increases in heat mitigation cost^11,12^.

People of color usually experience more heat exposure, higher vulnerability to heat, and lower adaptive capacity^13–15^. In the U.S., people of color are usually exposed to stronger surface urban heat island effects and higher moist heat stress than white people^16,17^. People of color are not only exposed to more heat but also more vulnerable to heat due to fewer heat adaptation strategies^18,19^. For example, black Americans are 40% more likely than others to live in areas with high heat-induced mortality^20^. Most previous studies examine heat exposure for one year at a coarse spatial scale, often census tract or block group^21,22^. Moreover, even though 40% of the U.S. population lives in small (50,000–100,000) and mid-size (150,000–500,000) cities, previous studies have mainly focused on large cities with populations of 500,000 or more. Urban heat inequalities and the change over time in mid-sized and small cities are less understood, even if these cities can have high proportions of people of color^23^. To the best of our knowledge, there is no study on inequality in heat exposure and adaptation over time that covers all the cities in our study area.

Cities can adapt to extreme heat through several strategies, such as tree planting, air conditioning, green roofs, and cool pavements. In this study, we focus on urban tree cover and air conditioning. Urban tree cover is an effective outdoor heat adaptation strategy^24,25^. Tree crowns can reduce solar radiation penetration, which lowers the ambient and surface temperatures in tree shades^26^. Furthermore, the evapotranspiration of trees absorbs heat from the atmosphere, decreasing the air temperature^27^. Many studies have shown that increasing tree canopy cover would reduce land surface temperature and air temperature^28^. For example, adding trees in all plantable areas in California would reduce the average urban land surface temperature by 1.8 °C and reduce heat-related medical visits significantly^29^; Increasing 10% of urban vegetation could reduce air temperature by up to 0.8 °C in Toronto^30^. However, predominantly people of color communities have disproportionately low tree cover compared to whiter areas^31–33^. There is little study on how tree cover and vegetation cover in predominantly people of color communities change over time.

Using air conditioning (AC) is an important indoor heat adaptation strategy^34^. AC use can reduce mortality, hospitalizations, and morbidity due to heat-induced illness^35,36^. However, there is high inequality of AC ownership in the United States as well as globally^37^^,^^38^. In San Diego, there was a 15% increase in hospitalizations during hot weather in neighborhoods with less AC access, whereas no significant increase was found in places with higher AC access^39^. The probability of AC ownership is lower in Black, Hispanic, or Latino Americans than in other race groups based on a census tract level analysis of 115 US metro areas^37^. However, the direct empirical and fine-scale evidence on the intra-urban differences in AC rate in different communities is scarce^37,40^. In summary, there is a lack of comprehensive studies of the relationship between heat exposure, tree cover, and AC ownership rate at a fine spatial resolution and long temporal periods in mid-sized and small cities.

**Fig. 1:**
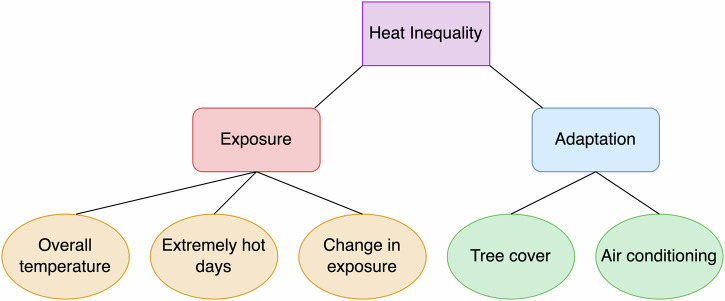
Conceptual figure of heat inequality.

In this study, we investigate heat inequality in different racial communities over time in the ten largest cities in Connecticut. These ten cities are the largest in Connecticut but are considered mid-sized or small in the U.S. (Fig. S1 and Table S1). We explore heat inequality in two categories, heat exposure and adaptation strategies (Fig. 1). Specifically, we examine three types of heat exposure inequalities: (1) overall temperatures, (2) extremely hot days, and (3) changes in exposure. We investigate two types of heat adaptation inequalities: (1) tree cover and (2) air conditioner ownership rates. We aim to answer the following research questions:

1. How were overall temperatures different in people of color (POC) and white people?
2. How many extremely hot days did people of color and white people experience over time?
3. How did heat exposure change over time for people of color and white people?
4. How are tree cover and AC ownership rates different for people of color and white people?

## Results

###  Increasing heat disproportionately affects POC

**Fig. 2:**
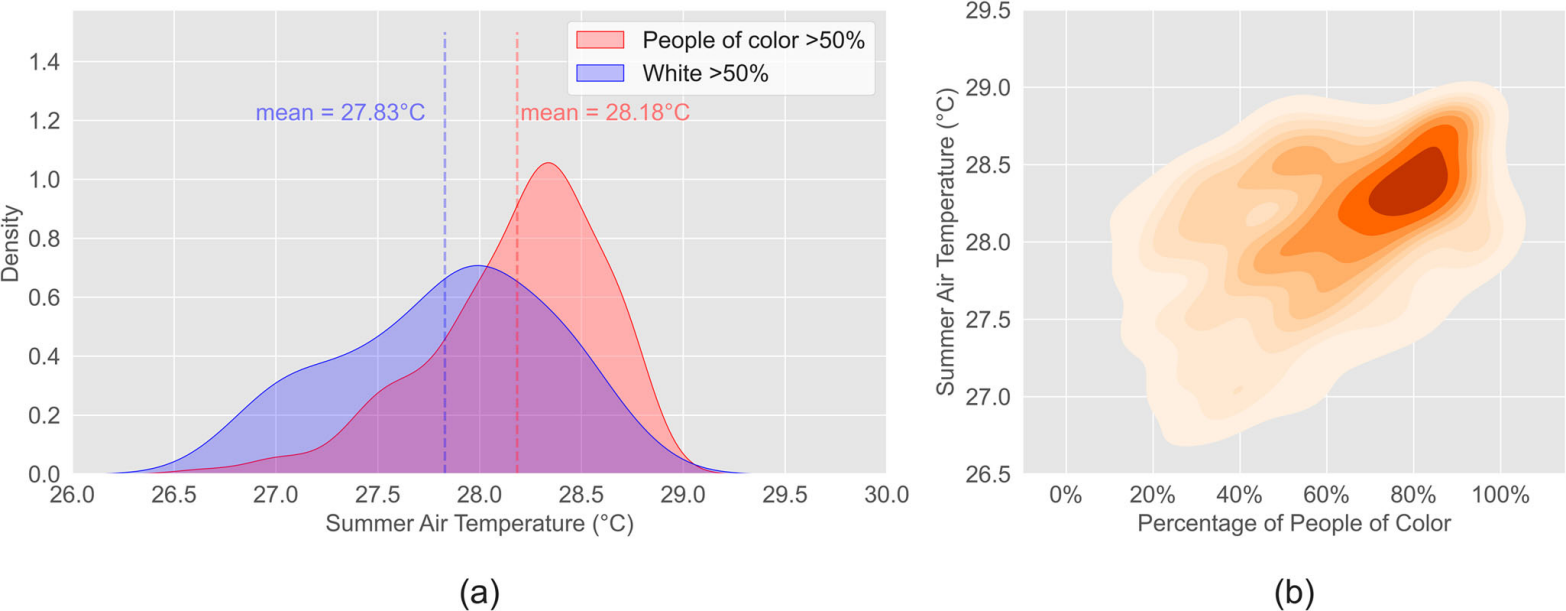
Inequity of air temperature of POC and white communities. (**a**) The mean air temperature in predominantly POC communities is 0.35 °C higher than predominantly white communities (*p* < 0.001); (**b**) Communities with higher POC percentages have higher air temperature (*p* < 0.001).

**Fig. 3:**
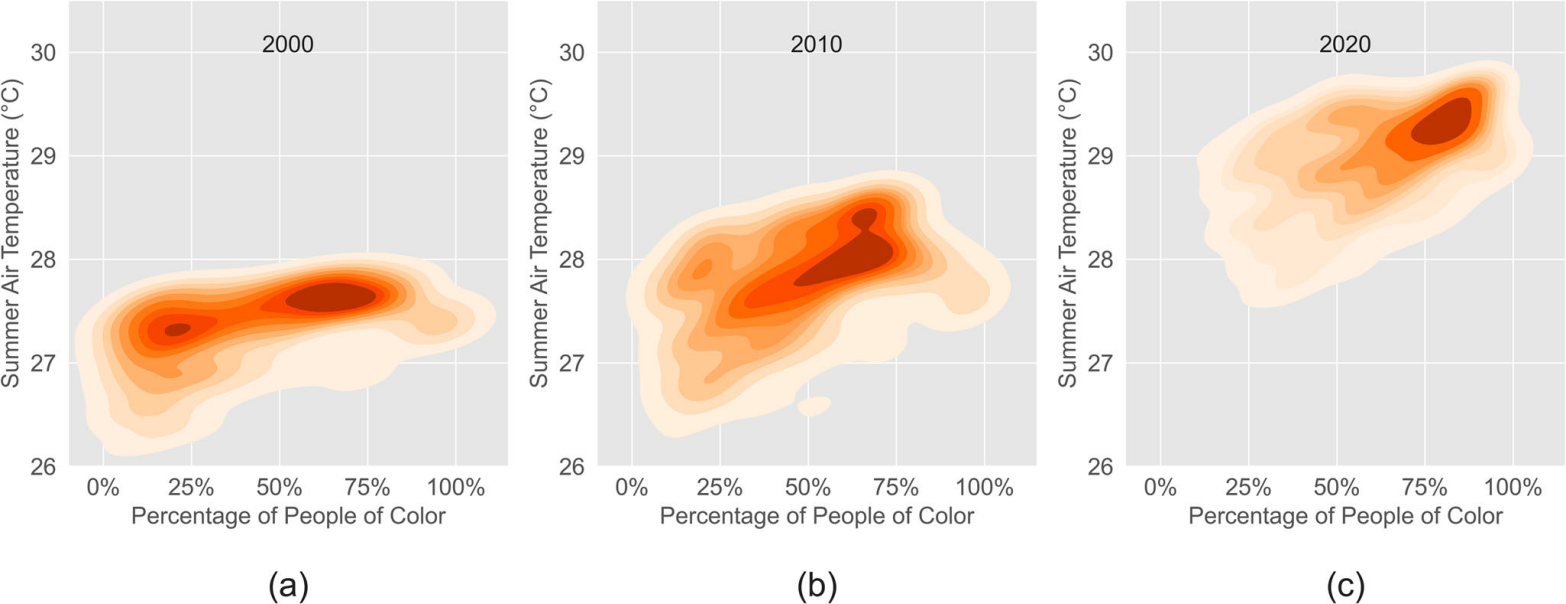
More POC communities have been exposed to high summer air temperatures over time. Summer air temperature and POC percentage in (**a**) 2000, (**b**) 2010, and (**c**) 2020. People of color communities had higher summer air temperatures in 2000, 2010, and 2020 (*p* < 0.001). The number of communities with high summer air temperatures has increased over time.

Increasing urban heat disproportionately affects POC. We analyzed the relationship between mean daily maximum air temperature in summer from 2003 to 2020 and race at the census tract level. The mean summer air temperature from 2003 to 2020 in predominantly people of color communities and predominantly white communities are 28.18 °C and 27.83 °C, respectively (Fig. 2). Predominantly POC communities have significantly higher air temperatures (*p* < 0.001). The summer air temperatures are significantly positively correlated with the percentage of POC (*p* < 0.001). The density plots in Fig. 3 show that the majority of blocks shifted from high percentages of white communities with low temperatures in 2003 to high percentages of POC communities with high temperatures in 2020. Moreover, we ran a non-spatial linear model and a spatial lag model between the summer air temperature and socioeconomic variables in 2020, including the percentage of people of color, the percentage of females, the percentage of people aged 65 or above, and median household income. Both models show that high percentages of POC are significantly associated with high summer air temperatures (Table S2).

**Fig. 4:**
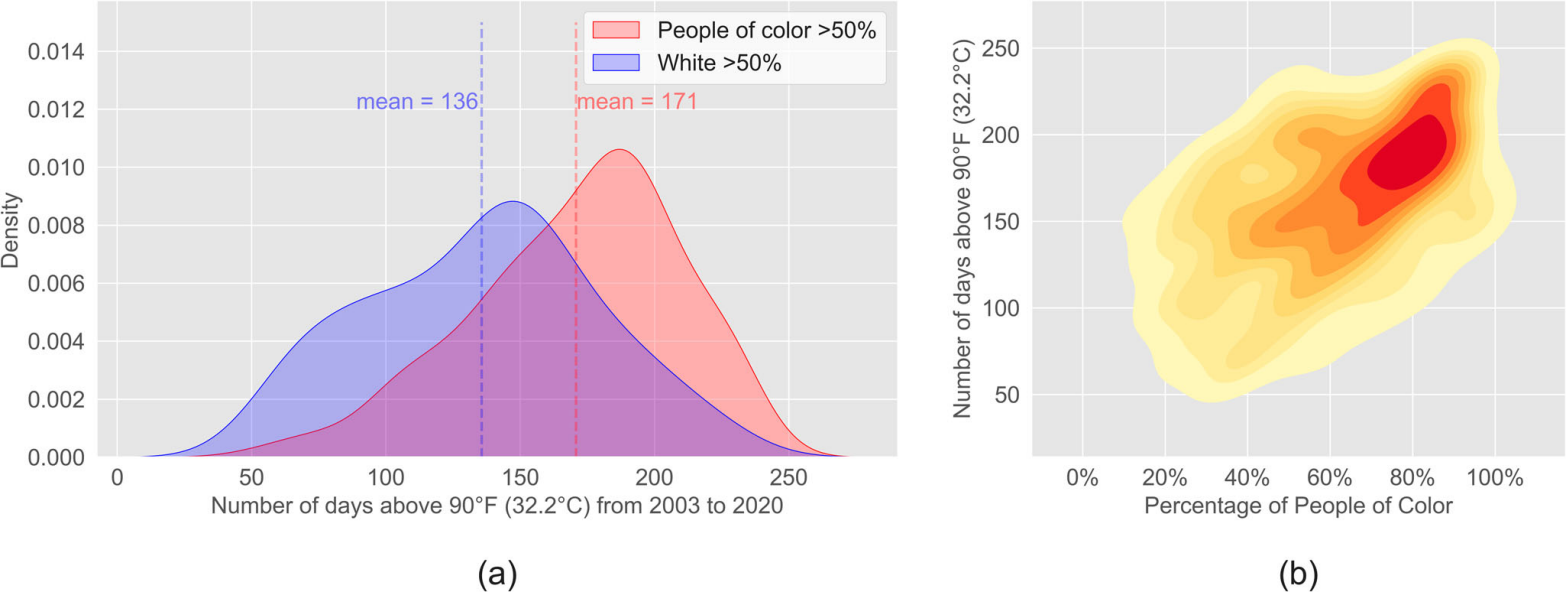
Inequity of number of extremely hot days (air temperature above 90 °F (32.2 °C)) from 2003 to 2020 between POC and white communities. (**a**) The mean air temperature in predominantly POC communities is 0.35 °C higher than predominantly white communities (*p* < 0.001); (**b**) Communities with higher POC percentages have higher air temperature (*p* < 0.001).

**Fig. 5:**
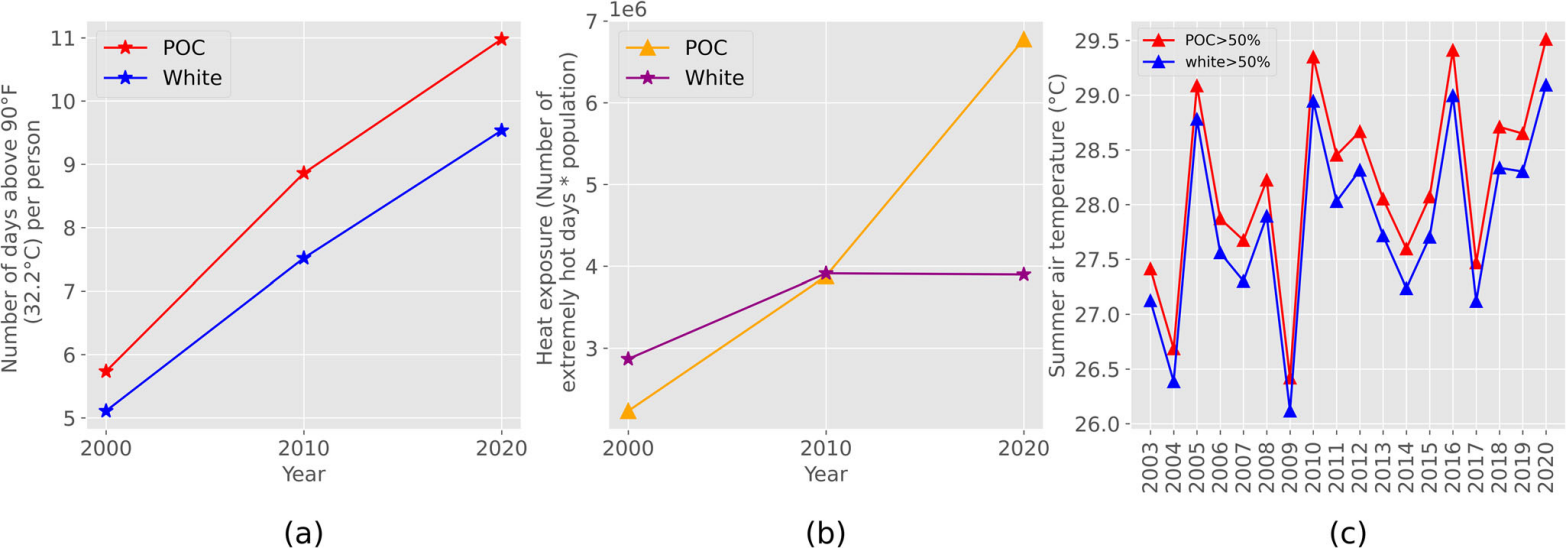
More extremely hot days and more heat exposure for POC. (**a**) Number of extremely hot days (air temperature above 90 °F (32.2 °C)) over time per POC and per white person; (**b**) Heat exposure, defined as the number of extremely hot days times population of POC and white, respectively; (**c**) Higher increase in summer air temperature for POC communities.

Furthermore, people of color experienced more extremely hot (air temperature above 90 °F (32.2 °C)) days and a larger increase in heat exposure. From 2003 to 2020, predominantly POC communities experienced 35 more extremely hot days than predominantly white communities, which is about 2 days per year on average (Fig. 4). The number of extremely hot days is significantly positively correlated with the percentage of people of color (*p* < 0.001). Although both people of color and white people experienced more extremely hot days over time, each person of color experienced a larger increase in the number of extremely hot days, which increased from 5.7 days in 2000 to 11.0 days in 2020, compared to 5.1 days in 2000 to 9.5 days in 2020 for each white person (Fig. 5a). Compared with white people, the heat exposure of people of color had significantly larger increases from 2000 to 2020 due to the increased number of extremely hot days and increased people of color population (Fig. 5b). From 2003 to 2020, the average summer air temperature increase in predominantly POC communities was 0.0749 °C per year, which is larger than the increase in predominantly white communities (0.0698 °C per year) (Fig. 5c).

**Fig. 6:**
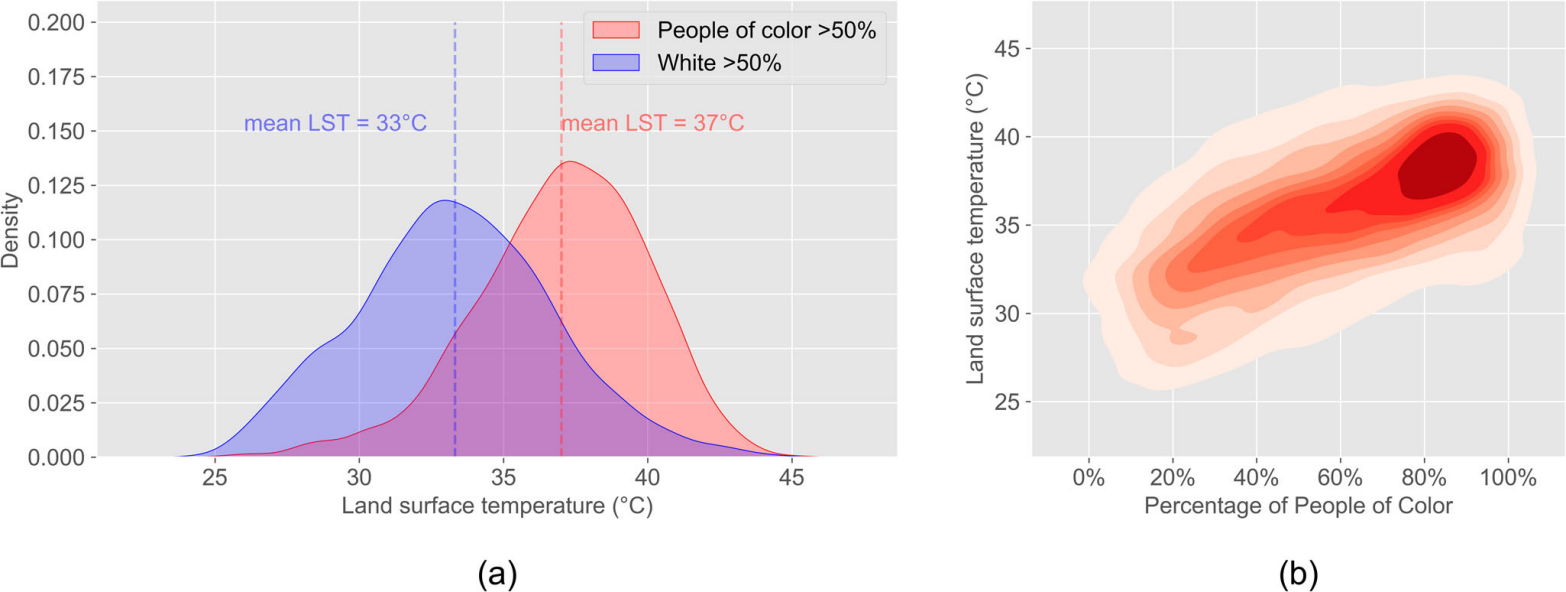
Inequity of LST between POC and white communities. (**a**) The mean LST in POC communities is 4 °C higher than white communities (*p* < 0.001); (**b**) Communities with higher POC percentages have higher LST (*p* < 0.001).

An analysis of all census blocks for the top ten most populous cities in Connecticut shows that land surface temperature (LST) is significantly higher in predominantly POC communities than in predominantly white communities. Predominantly POC communities experience a mean LST that is 4 °C higher than predominantly white communities. The 30-year mean LST in predominantly white communities is 33 °C, whereas in predominantly POC communities, it is 37 °C (Fig. 6a). Land surface temperature is positively correlated with the percentage of people of color (Fig. 6b). For block-level analysis of each city, the mean LST is significantly higher in predominantly POC communities than in predominantly white communities for all cities. The disparity of mean LST between predominantly POC communities and predominantly white communities ranges from 1.1 to 5.1 °C (Table S3). The disparities in LST increased for seven cities and decreased for three cities from 1990 to 2020. For nine out of ten cities, LSTs in 1990, 2010, and 2020 are significantly and positively correlated with the percentage of people of color (Table S4).

**Fig. 7:**
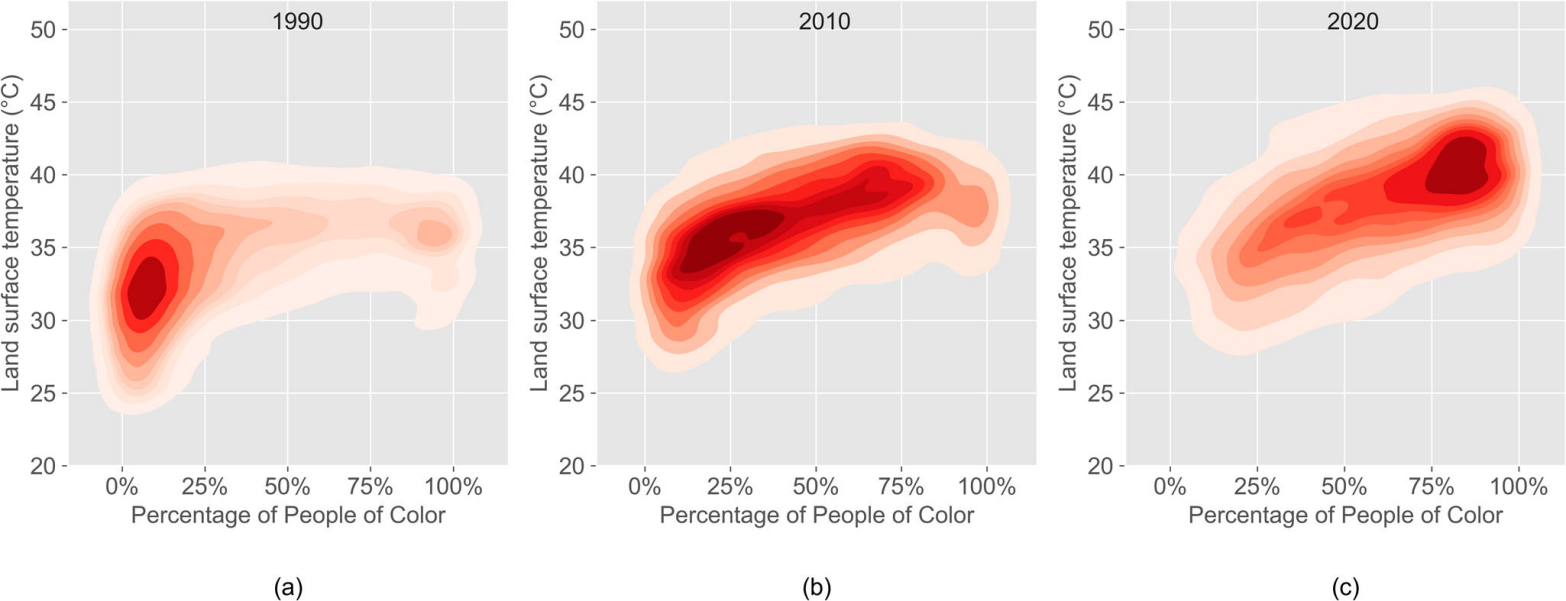
More POC communities have been exposed to high LST over time. LST and POC percentage in (**a**) 1990, (**b**) 2010, and (**c**) 2020. People of color communities experienced higher LST in 1990, 2010, and 2020 (*p* < 0.001). The number of communities with high POC has increased over time.

The state-level analysis of LST and POC percentages in 1990, 2010, and 2020 shows that predominantly POC communities have experienced higher temperatures than predominantly white communities in all studied years (Fig. 7). Although the cities have become more diverse since 1990 (the percentage of POC residents has increased), the disparity between predominantly POC and predominantly white communities persisted in the last three decades. The disparity between predominantly POC communities and predominantly white communities of mean LST increased slightly over time based on block-level analysis of all cities. Moreover, the majority of blocks shifted from high percentages of white communities with low LST in 1990 to high percentages of POC communities with high LST in 2020.

We analyzed the correlation between LST and the percentage of American Indian/Alaska Native alone, Black/African American alone, Hispanic/Latino alone, Asian/Pacific Islander alone, and people of mixed races, respectively, in 1990, 2010, and 2020. For all years, the percentage of people of mixed races has a stronger relationship with LST than those of one non-white race alone. However, this relationship is slightly weaker than that of LST and the percentage of people of color. Among all single non-white racial groups, the percentage of Black or African American has the strongest positive correlation with LST in all years.

Our results suggest that people of color in Connecticut’s ten most populous cities are not only disproportionately affected by hot temperatures, but these communities are also getting hotter over time. All these findings suggest that people of color have been exposed to more heat and will likely experience more heat exposure in the future.

### Low air conditioning ownership rates in POC communities

**Fig. 8:**
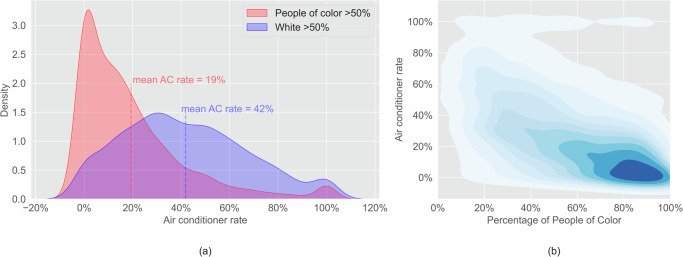
Inequity of AC ownership rates between predominantly POC and predominantly white communities. (**a**) The mean AC rate in predominantly POC communities is 23% lower than in predominantly white communities (*p* < 0.001); (**b**) Communities with higher POC have lower AC rates (*p* < 0.001).

One important cooling option is air conditioning (AC). Usage of AC would significantly lower the risk of increasing heat exposure in POC communities. However, we found that the AC ownership rate in 2022 (the percentage of buildings with air conditioning per block) is lower in predominantly POC communities than in predominantly white communities. Based on a block-level analysis at the state level, we find that the mean AC rate in predominantly POC communities is 23% lower than in predominantly white communities. (Fig. 8a). A block-level analysis of all cities shows that AC ownership rates are negatively correlated with the percentage of people of color (Fig. 8b). AC rates are significantly lower in predominantly POC communities in all cities. The disparity of the AC rate between predominantly POC and predominantly white communities ranges from 4% to 29%, and in eight out of ten cities, the disparity is equal to or higher than 15% (Table S5). All cities show a negative relationship between the AC rate and the percentage of POC residents per block. In nine out of ten cities, this relationship is statistically significant (*p* < 0.01).

**Fig. 9:**
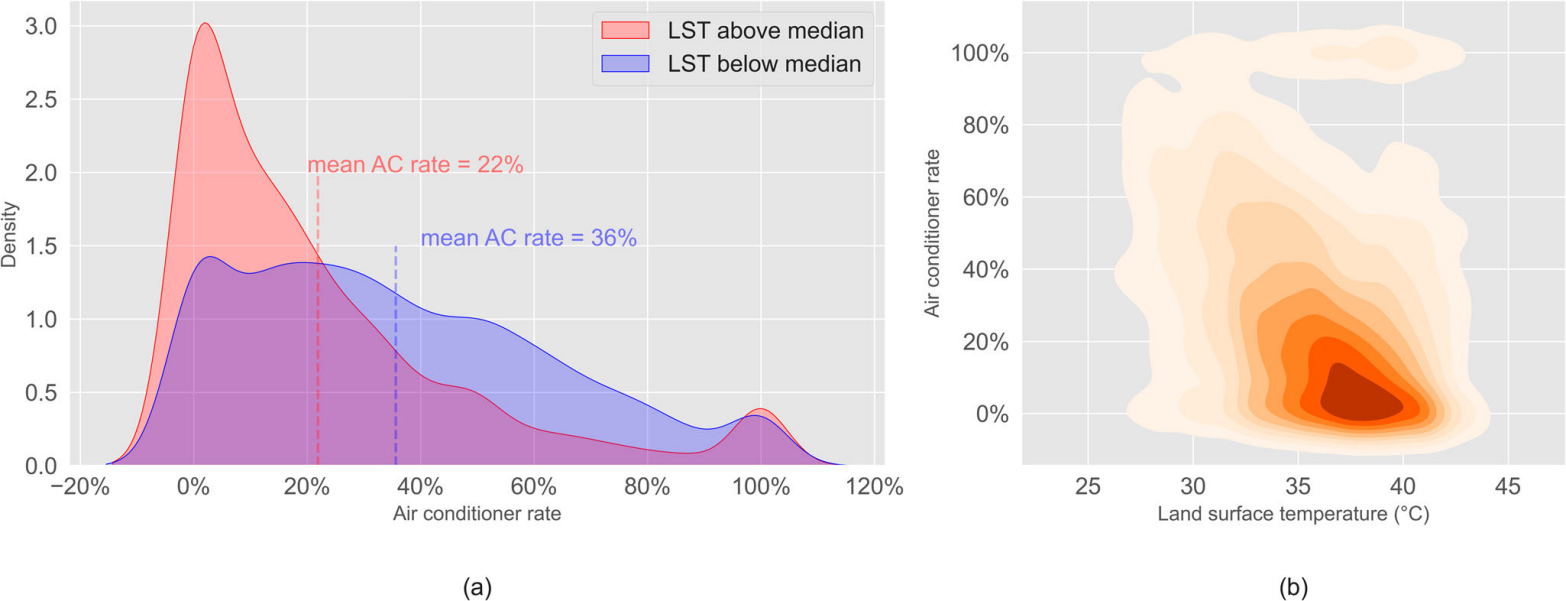
Inequity of AC rates in communities with high LST. (**a**) The mean AC rate in communities with LST above the median is 14% lower than communities with LST below the median (*p* < 0.001); (**b**) Communities with higher LST have lower AC rates (*p* < 0.001).

Furthermore, AC ownership rates are lower in communities with higher LST. In blocks with LST below the statewide median across these ten cities, the mean AC rate is 36%, whereas the mean AC rate is 22% in the blocks with LST above the median (Fig. 9a). AC rate is negatively correlated with LST (Fig. 9b). This indicates that hotter communities have fewer air conditioners. The communities with high LST and low AC access are particularly vulnerable to increasing heat exposure.

### Low tree canopy cover in POC communities

**Fig. 10:**
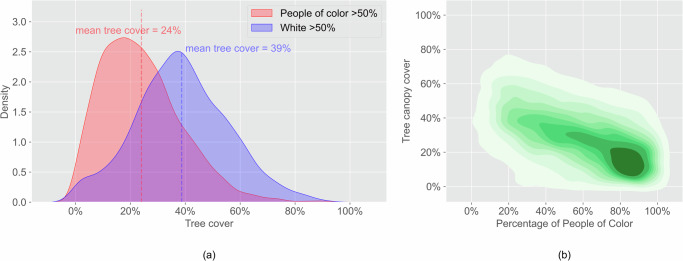
Inequity of tree cover between predominantly POC and predominantly white communities. (**a**) The mean tree cover in predominantly POC communities is 15% lower than in predominantly white communities (*p* < 0.001); (**b**) Communities with higher POC percentage have lower tree cover (*p* < 0.001).

Another important cooling strategy for cities is tree canopy cover. However, tree cover is lower in predominantly POC communities than in predominantly white communities. For all cities, the mean tree canopy cover in predominantly white communities is 39%, whereas in predominantly POC communities, it is 24% (Fig. 10a). For block-level analysis across all cities, tree cover is negatively correlated with percentage of POC (Fig. 10b). A block-level analysis of each city showed that both the disparity in tree cover and vegetation cover in predominantly POC and predominantly white communities ranges from 2% to 22%, and the disparity is significant in nine out of ten cities (Table S5). The disparity in vegetation cover ranges from 0% to 21% and is significant in nine out of ten cities. In nine out of ten cities, tree cover and percentage of POC have negative and significant correlations (Table S6). Vegetation cover shows a similar pattern.

**Fig. 11:**
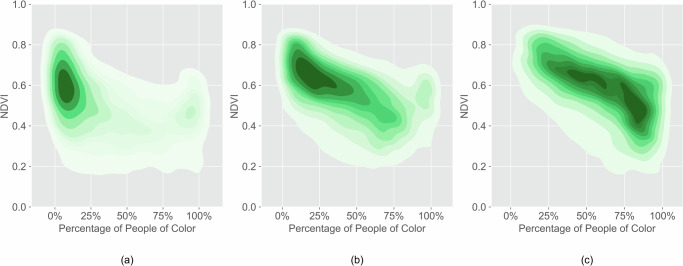
Predominantly people of color communities have lower NDVI in all studied years. NDVI and POC percentage in (**a**) 1990, (**b**) 2010, and (**c**) 2020.

Predominantly POC communities consistently had lower NDVI than predominantly white communities in 1990, 2010, and 2020 (Fig. 11). The majority of the blocks shifted from high NDVI with low percentage of POC communities to low NDVI with high percentage of POC communities (Fig. 11). Block-level analysis of each city shows that disparity in NDVI between predominantly POC communities and predominantly white communities is significant (Table S7). NDVI is negatively correlated with the percentage of POC for nine out of ten cities in 1990, 2010, and 2020 (Table S8). Disparities in NDVI increased in half of the studied cities. With low tree canopy cover and low AC rate, POC communities have higher risks of heat-induced illness and mortality than white communities.

We also analyzed the correlation between NDVI and the percentage of American Indian/Alaska Native alone, Black/African American alone, Hispanic/Latino alone, Asian/Pacific Islander alone, and people with mixed races, respectively, in 1990, 2010, and 2020. For all years, the percentage of people of mixed races has stronger negative relationships with NDVI than those of one non-white race alone. Among all single non-white racial groups, the percentage of Black or African American has the strongest negative correlation with NDVI for all years.

### Increasing tree canopy cover to mitigate heat

We found that NDVI change is significantly and negatively correlated with LST change in all cities (Table S9), which indicates higher tree canopy cover leads to lower temperature. These results indicated that planting trees can mitigate temperature increases. However, we also found that most blocks have experienced increases in LST and NDVI. This indicates that over the last three decades, the increase in vegetation cover was not enough to completely offset the increase in urban heat.

In communities with high POC percentages, LST is high whereas tree cover and AC rates are both low. This suggests that POC communities are exposed to more heat and more vulnerable to extreme heat. With lower air conditioning rates and low tree canopy cover, POC communities have less adaptation capacity to high temperatures, compared to white communities. This highlights the importance of increasing tree canopy as an adaptation strategy to high temperatures. Among the ten cities, cities with the highest disparity in LST between predominantly POC and predominantly white communities, such as Stamford, Danbury, and Norwalk, also have high disparities in AC rate and vegetation cover (Fig. S3).

In summary, we found that mean land surface temperature is significantly higher in predominantly POC communities in all cities. We also found that heat exposure in predominantly POC communities increased over time, not only because of increasing temperature but also the increase of the percentage of POC. Vegetation covers in predominantly POC communities were consistently lower than in predominantly white communities over time. The AC rate in predominantly POC communities is significantly lower than in predominantly white communities in all cities. With increasing heat exposure, planting trees is important in predominantly POC communities, especially for those with low AC access. The time series analysis shows that increasing vegetation cover can mitigate the increase of heat. However, the current degree of vegetation cover increase is not high enough to completely offset the temperature increase. These findings highlight the importance of increasing tree canopy cover in POC communities to mitigate heat, especially in communities with low AC access.

## Discussion

In this study, we investigated heat exposure and vegetation cover in different communities over time, and intra-urban variations of AC ownership in the ten largest cities in Connecticut, using multi-decade satellite data, census data, high-resolution airborne imagery, and building-level AC data. We find that predominantly people of color communities in Connecticut face inequalities in heat exposure and adaptation options. First, predominantly POC communities have been exposed to both higher air temperatures and land surface temperatures. Also, each person of color experienced more extremely hot days than each white person and the disparity has increased over time. Second, heat exposure of POC has a larger increase than that of white people, due to increased population of people of color and temperature rise. People of color population increased over time in most studied cities, and they are more likely to live in places with high temperatures. This suggests that more action is needed to mitigate increased heat exposure in POC communities. Third, predominantly POC communities have lower AC ownership rates and constantly have lower tree cover and vegetation cover. Without effective heat adaptation strategies, heat morbidity and mortality could sharply increase in predominantly POC communities in the future.

The results have several implications for policymakers. First, increasing heat adaptation strategies for underserved communities is essential to mitigate heat inequality. The two heat adaptation strategies we studied here, planting trees and air conditioning, have different effects on heat at the neighborhood scale and the timescale of impact is different. Running AC could increase outdoor temperatures and heat exposures in the neighborhood, whereas planting trees can mitigate heat at the neighborhood level. Regarding the timescale of impact, planting trees is a slow adaptation strategy with long-term co-benefits such as reducing AC energy use in buildings due to shading. It usually takes several years for newly planted trees to grow and become effective for heat adaptation. Trees can provide increasing cooling benefits over time and the benefits can last for more than a hundred years. Moreover, trees provide other ecosystem services, such as improving air quality and reducing stormwater runoff. Air conditioning can provide immediate cooling but can slowly degrade over time and does not provide other co-benefits.

Second, our study suggests that planting trees is an important heat mitigation and adaptation strategy for predominantly POC communities that are getting hotter and with low AC ownership rates. Urban temperatures are expected to continue to increase, and extreme heat waves will become more frequent. It is expected that urban heat exposure in predominantly POC communities will substantially increase. Limited cooling options and increased heat exposure could lead to an increase in heat-induced morbidity and mortality in predominantly POC communities. Because predominantly POC communities are getting hotter and have lower tree cover, tree-planting initiatives should prioritize communities with low tree cover, high temperatures, and low AC rates. To better implement tree planting initiatives in these communities, it is important to enhance community engagement in tree planting, maintenance, and stewardship. Some good examples are the MillionTrees program in New York City and Los Angeles^41^, and the Urban Resources Initiative (URI) in New Haven^42^. Future studies should investigate canopy increase potential and tree species selection to maximize heat mitigation benefits of tree planting initiatives. Previous tree-planting initiatives usually did not account for equity in urban forests^43^. In the future, tree-planting initiatives should take environmental justice into account. Another important consideration is that planting trees can also increase humidity and may increase the wet-bulb temperature in some regions^44^. Future research should consider the effect of planting trees on both temperature and humidity in different climate zones.

Third, environmental justice should be taken into consideration in future policymaking^45^. Environmental justice is defined as the “*fair treatment and meaningful involvement of all people regardless of race, color, national origin, or income*”, according to the Environmental Protection Agency^46^. One of the key challenges is to define and identify environmental justice communities, which can provide a legal foundation for policy interventions and facilitates the allocation of resources and funds to these communities. There are varying definitions across state and federal agencies. In January 2021, the Biden Administration issued a new executive order creating the White House Climate and Economic Justice (WHCEJ) Screening Tool^23,47^. The tool uses socioeconomic and environmental data to identify communities at the census tract level that are economically disadvantaged and overburdened by pollution and underinvestment in housing, infrastructure, and health care at census tract level^23^. In Connecticut, the state’s Department of Energy & Environmental Protection (DEEP) defines environmental justice communities purely on economic status at the census block group level. One notable limitation is that both tools do not consider race, although race is an important variable in environmental justice, as shown in this study and other studies^48^. Furthermore, the tools do not directly include a heat exposure and vulnerability component, which highlights the importance of more focused geospatial analysis on inequality in heat exposure and vulnerability at state and city levels^12,49^.

Fourth, this study underscores the value of multi-temporal and multi-source geospatial data to identify the urban heat threats that POC communities face. The study provides new geospatial datasets that can be used to identify heat-vulnerable communities and prioritize tree-planting areas that would yield the greatest benefits for heat reduction and environmental justice (Fig. S4). A recent bill in the Connecticut General Assembly aims to plant more trees in the five largest cities and environmental justice communities in the state^50^. The bill requires increasing the percentage of the tree canopy of environmental justice communities to a level of at least five percent before Jan 2024^50^. The products from our study can provide geospatial information to guide the implementation of this bill. Furthermore, the study demonstrates the importance of fine-scale datasets of heat exposure and access to adaptation strategies. Building-level AC data is particularly important for identifying heat-vulnerable communities. However, fine-scale AC dataset is limited in location and usually not open to the public. In the context of global warming and increasing heat exposure, open access AC data with fine-scale resolution is essential to heat adaptation studies and decision-making^51^.

There are several limitations of our study, and future research can address these limitations. First, we only used AC ownership as an important indicator of heat adaptation capacity. However, other heat adaptation strategies are not included in our analysis^52,53^. This is because we consider AC to be one of the most important heat adaptation strategies with publicly available datasets. In the future, more indoor cooling options should be explored if data is available. Moreover, AC ownership is not equivalent to AC usage. Due to the cost of running AC, AC usage could be quite different in different communities even if the AC ownership rate is similar. Future studies should investigate possibleinequalities of AC usage in different communities. Also, our AC data is based on property assessment, which may systematically underestimate window AC, especially in buildings with high percentages of renters. We used the data in this study because the focus of this study is on the disparities of AC rate, and it is the best available open-access data. Future studies can investigate intra-urban AC disparities if more accurate AC data is available. Second, we did not consider mobility in heat exposure and adaptation options. For example, heat exposure is different for people who travel between the workplace and home and those who do not. Also, people who work indoors and those who work outdoors have substantially different heat exposure. Future research should take mobility and working environment into consideration. Third, this study was conducted only for the ten largest cities in Connecticut. It is unknown whether other mid-sized cities have similar patterns. Future research can expand this study to more cities across the United States.

## Methods

Our study area is in the State of Connecticut, located in the Northeast of the United States (Fig. S1), which is a region with the largest disparities in heat exposure and tree cover according to ref. ^32^. The study area is the top ten largest cities by population in Connecticut, including Bridgeport, Stamford, New Haven, Hartford, Waterbury, Norwalk, Danbury, New Britain, Hamden, and Manchester^54^. These cities contain a majority of the environmental justice communities in the state according to the White House Climate and Economic Justice^23^ and the State of Connecticut Environmental Justice mapping tools^55^. These cities are home to approximately one-third of the state’s total population but contain 70% of the people of color and 52% of the low-income residents in the state^54,56^. Table S1 includes the basic demographic statistics of these cities. Data used in this study includes US census data in 1990, 2000, 2010, and 2020, land use parcel data, building-level property assessment data, satellite data, airborne data, and OpenStreetMap (OSM) data.

### Race

Race data at census block level in 1990, 2010, and 2020 was from the United States Census. The data was downloaded from IPUMS (Integrated Public Use Microdata Series). Percentage of people of color was calculated as the population of people of color divided by the total population of each block in our study area. The percentage of POC is equal to one minus the percentage of white alone. If the percentage of POC is larger than 50% in a block, the block is categorized as a predominantly POC community; otherwise, it is categorized as a predominantly white community. We also calculated the percentage of the population of different racial groups, including White alone, American Indian/Alaska Native alone, Black/African American alone, Hispanic/Latino alone, Asian/Pacific Islander alone, and people with mixed races. In the open-access census data, race is the only demographic variable where the block-level data is available across different years.

### Air temperature and land surface temperature

We used a global daily near-surface air temperature dataset from 2003 to 2020 at 1 km resolution^57^. This dataset was developed by combining ground-station-based measurements and satellite observations^57^. It has been used in other urban heat island studies^58^. We calculated the mean daily maximum air temperature in summer (June to September) at the census tract level.

Furthermore, we used Landsat Collection 2 land surface temperature for time series analysis of urban heat in our study area. The spatial resolution of the resampled LST products in Landsat Collection 2 is 30-meter resolution (The original resolution of LST is 60 m, 100 m, or 120 m). The cloud and shadow observations were removed using the Landsat Quality Assessment band. Only summer (June to September) observations were used, because using summer observations results in better model fitting than using whole-year observations and only summer LST is relevant to this study. We ran a time series model, Continuous Change Detection and Classification (CCDC)^59^, with land surface temperature on Google Earth Engine (Fig. S2). CCDC uses harmonic models to predict the next observations. If the next observation significantly deviates from the predictions, the CCDC model will detect a change, the current model will break and a new model will be initiated^59^. The benefit of using the CCDC model is that both the abrupt change and gradual change can be captured^60,61^ while eliminating the effect of noises and seasonality. Furthermore, CCDC can provide continuous predictions of any given date, called synthetic values^62^. Here, we calculated the CCDC model prediction for July 1st (synthetic values) every year from 1990 to 2022. Choosing model prediction for the same date every year ensures that the values are comparable in different years and less affected by data availability on different dates^61^. July 1st was chosen because it is in the middle of summer and is during the hottest period. We also tested using July 31^st^ and found that the disparities and correlations are slightly smaller and thus we finally used July 1^st^ for our analysis. We produced annual land surface temperature maps from 1990 to 2022 for our study area. The land surface temperature maps were aggregated at the block level.

### AC ownership rate

Block-level AC ownership rate was calculated from building-level AC availability for each block. CAMA (computer-assisted mass appraisal) data and land use parcel data in the state of Connecticut in 2022 provide AC availability at parcel level (A parcel usually includes one building and surrounding open area). 2022 is the only year so far when comprehensive CAMA data in Connecticut is available. Building footprints in Open Street Map were spatially joined with the parcel-level data to acquire building-level AC availability. AC rate at the block level was calculated as the number of buildings with AC divided by the total number of buildings.

### Tree cover and vegetation cover

High-resolution (60 cm) airborne imagery of NAIP (National Agriculture Imagery Program) for the state of Connecticut was used to derive tree cover maps. The imagery was acquired in the summer of 2018, which is the most recent available high-resolution imagery in Connecticut. For each city, at least 1000 training points of land cover were collected. The land cover classes included trees, grass, buildings, paved, water, barren, and shadow. In total, 14,238 training points were collected. The red, green, blue, and near-infrared (NIR) bands were used in the classification. Object-based classification was conducted in Google Earth Engine. The first step is image segmentation using super-pixel clustering based on SNIC (Simple Non-Iterative Clustering). Spectrally similar and spatially close pixels are clustered into objects. The second step is using the training data and machine learning (random forest) to classify the objects, using the mean band values (red, green, blue, and NIR, respectively), standard deviation, area, perimeter, width, and height of each object. After automated classification, we manually corrected misclassification in several places with complex land cover, such as wetlands. The land cover maps were aggregated into tree cover maps and vegetation cover maps at 60 cm. Pixel-level accuracy assessment was conducted for each city using 300 simple random sample units (3000 in total). Our tree cover and vegetation cover maps achieved high accuracy. The overall accuracies of tree cover maps range from 87% to 93% (Table S9). The producer’s and user’s accuracies of tree cover maps range from 80% to 96%. The overall accuracies of vegetation cover maps range from 91% to 97%. The producer’s and user’s accuracies range from 85% to 99%. In New Haven, we also compared the map with field inventory data of street trees^42^. 97% of street tree crowns are correctly mapped within a 3-meter buffer of street tree locations in field inventory data. Block-level tree cover and vegetation cover were calculated from the high-resolution tree cover maps.

### Normalized difference vegetation index

NDVI was used as a proxy of vegetation cover over time. We used Landsat Collection 2 from 1990 to 2022 for time series analysis of NDVI. Only summer clear observations were used. The cloud and shadow observations were removed using the Landsat Quality Assessment band. We ran the CCDC model with NDVI on Google Earth Engine and calculated the synthetic values on July 1^st^, similar to method Section 4.2. We produced annual 30-meter NDVI maps from 1990 to 2022 for our study area. The mean NDVI from 1990 to 2022 was also calculated for every pixel in our study area. Since our analysis was conducted at the block level, the 30-meter NDVI maps from 1990 to 2022 were aggregated at the block level.

### Disparity analysis

The disparity analysis was conducted at two spatial scales, census tract level and census block level. At the census tract level, we calculated the annual mean summer air temperature from 2003 to 2020 and compared the air temperature differences between predominantly POC and predominantly white communities. In the trend analysis, we fitted linear regression models with years and mean summer air temperatures to get the annual increases. In the decadal analysis, to match the census data and air temperature data, we used air temperature in 2003 and census data in 2000 for the analysis in 2000. We used the census data in 2010 and the mean summer air temperature of 2009 and 2010 for analysis in 2010. In the analysis of 2020, we used the census data in 2020 and mean summer air temperature in 2019 and 2020. We calculated the correlation coefficients between the air temperature at the percentage of POC. We also ran a non-spatial multivariate linear model and a spatial lag model^63^ between the summer air temperature and socioeconomic variables, including the percentage of people of color, the percentage of females, the percentage of people aged 65 or above, and median household income. Furthermore, we calculated the number of extremely hot days, defined as days with the summer air temperature above 90 °F (32.2 °C)^64^. We compared the number of extremely hot days experienced by each person of color and each white person. We also calculated the heat exposure, defined as the number of extremely hot days multiplied by the population of POC and white, respectively^2^.

At the census block level, we calculated the differences in mean LST (Method 4.3) in predominantly people of color communities and predominantly white communities by city and for the whole state, respectively. The distribution of the block-level LST of predominantly POC communities and predominantly white communities was plotted. The correlation coefficients between POC percentage and LST were calculated at the city level and state level, respectively. For inequality in AC ownership, we calculated the difference in mean AC rate between POC communities and white communities for all the blocks in all cities and in each city. The correlation coefficients between POC percentage and AC rate were calculated. Similarly, the disparity of tree cover and vegetation cover was calculated as the mean differences, and correlation coefficients with POC percentage were calculated. For vegetation cover over time, we calculated NDVI mean differences (Method 4.5) over time for predominantly POC communities and predominantly white communities.

## Supplementary information

Supplementary_materials_2024_0812

## Data Availability

All source data and generated data products in our study are publicly available with free access. The geospatial data products and a visualization tool can be accessed via https://github.com/shijuanchen/HEAT. The source data can be downloaded from the following links: The parcel data and property assessment data (CAMA) for the state of Connecticut can be downloaded from https://portal.ct.gov/datapolicy/gis-office/parcels-and-cama?language=en_US. The OSM data can be downloaded from https://data.humdata.org/dataset/hotosm_usa_connecticut_buildings. The census data can be downloaded from https://www.nhgis.org/. The Landsat data is available on Google Earth Engine: https://developers.google.com/earth-engine/datasets/catalog/landsat. The high-resolution airborne data (NAIP) is available on Google Earth Engine: https://developers.google.com/earth-engine/datasets/catalog/USDA_NAIP_DOQQ.

## Code availability

Code in this study will be available upon request.
