# Supplementary material for: More extremely hot days, more heat exposure and fewer cooling options for people of color in Connecticut, U.S
Source: NPJ Urban Sustain. 2024 Nov 3;4(1):47. doi: 10.1038/s42949-024-00186-5 (PMC11532198; doi:10.1038/s42949-024-00186-5)
Supplement: Supplementary file 1 — Supplementary_materials_2024_0812 [file 42949_2024_186_MOESM1_ESM.pdf]

More extremely hot days, more heat exposure and fewer cooling options for people of color in Connecticut, U.S.

Shijuan Chen*, Katie Lund, Colleen Murphy-Dunning, Karen C. Seto

* Corresponding author: shijuan.chen@yale.edu

Supplementary materials:

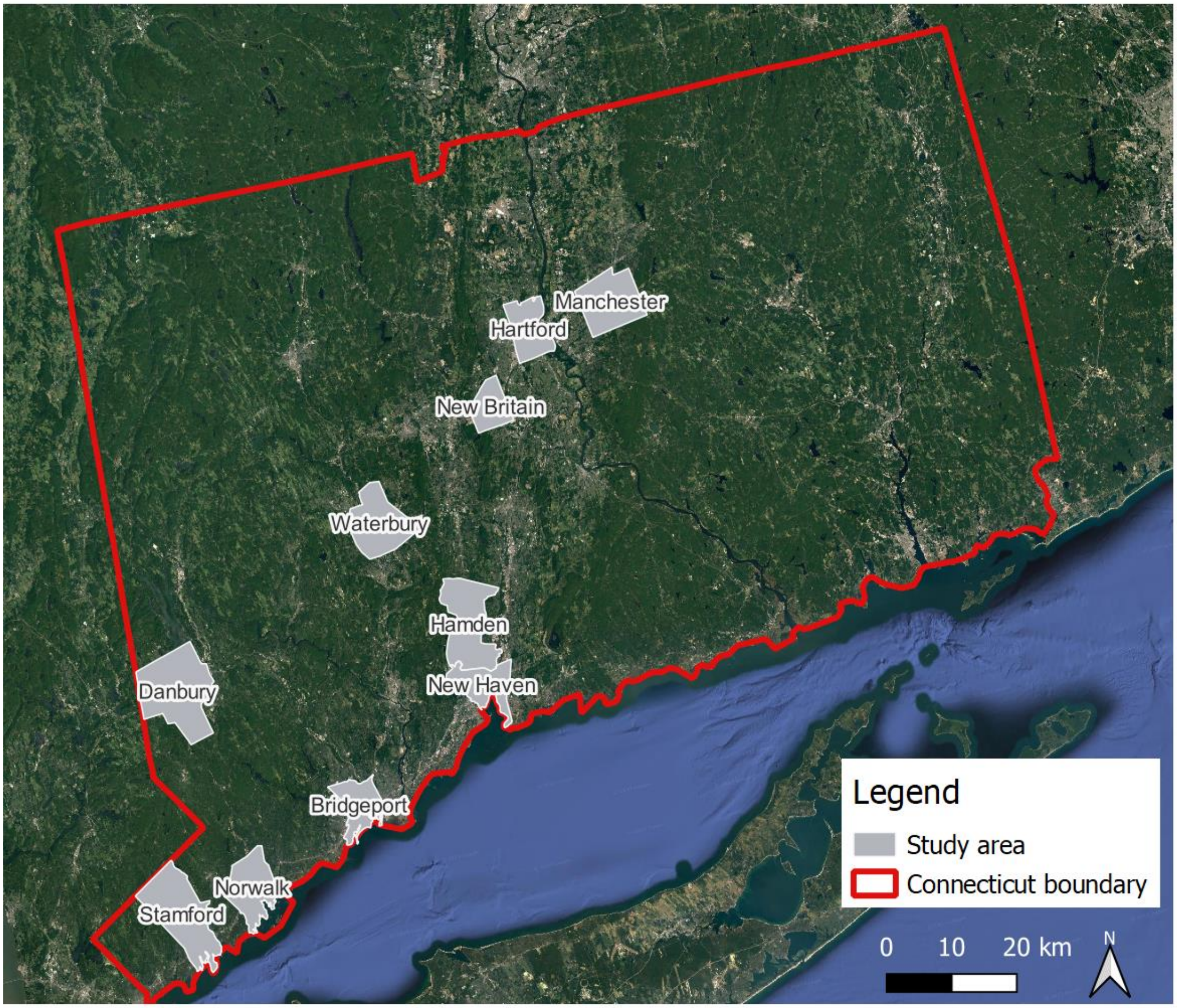

Figure S1. Study area - the ten largest cities in Connecticut, United States.

Table S1. Basic statistics of the ten largest cities in Connecticut.

City	Total Population	People of Color %	People Below the Poverty Line %	Elderly (Over 65) %
Bridgeport	150,764	66	23.2	12.1
New Haven	140,446	59.9	24.6	10.5
Stamford	130,830	40.7	9.4	15.3
Hartford	126,656	72.2	28.4	11.5
Waterbury	115,126	42.8	21.7	14.4
Norwalk	88,797	34.5	9.7	15.5
Danbury	87,490	41.9	10.6	15.1
New Britain	76,017	41.3	20.5	13.3
Hamden	65,986	44.2	9.3	16.2
Manchester	63,457	39.4	13.7	15.4

Table S2. Non-spatial linear model and spatial lag model summary. The dependent variable is the summer air temperature in 2020. The independent variables are the percentage of people of color (POC), the percentage of females (female), the percentage of people aged 65 or above (Elderly), and the median household income (Income).

Dependent	Air temperature	Model type	Non-spatial linear model	R-squared:	0.2897
Independent	Coefficient	Std.Error	z-Statistic	P value	Significance
Intercept	30.2539627	0.6455228	46.8673826	0.0000000	***
POC	1.2391737	0.1978081	6.2645241	0.0000000	***
Female	-3.1441803	1.2347245	-2.5464631	0.0114708	*
Elder	-0.4761703	0.5421087	-0.8783667	0.3805716	-
Income	0.1121232	0.1019711	1.0995588	0.2725614	-
Dependent	Air temperature	Model type	Spatial lag model	Pseudo R-squared:	0.8834
Independent	Coefficient	Std.Error	z-Statistic	P value	Significance
Intercept	4.0780451	1.4989354	2.7206276	0.0065158	**
POC	0.2295171	0.0989969	2.3184264	0.0204262	*
Female	-1.151262	0.5179545	-2.2227086	0.0262355	*
Elder	-0.4409641	0.2220054	-1.9862770	0.0470026	*
Income	-0.0659366	0.0429469	-1.5353049	0.1247090	-
Weight	0.8798873	0.0495961	17.7410687	0.0000000	***

Table S3. Disparities in LST (°C) between people of color communities and white communities over time (*p-value <0.05, **p-value<0.01, ***p-value<0.001).

City	LST mean	LST 1990	LST 2010	LST 2020
Bridgeport	2.5 ***	1.7 ***	2.0 ***	2.4 ***
Danbury	4.1 ***	4.3 ***	2.7 ***	4.5 ***
Hamden	3.4 ***	2.8 ***	3.3 ***	3.4 ***
Hartford	1.1 **	-0.2 -	0.7 **	0.8 -
Manchester	2.0 ***	5.4 -	2.4 ***	2.3 ***
New Britain	1.9 ***	1.1 **	1.3 ***	1.8 ***
New Haven	1.1 ***	2.0 ***	1.2 ***	1.0 ***
Norwalk	4.0 ***	3.6 ***	4.0 ***	4.3 ***
Stamford	5.1 ***	3.8 ***	4.7 ***	5.5 ***
Waterbury	2.1 ***	2.7 ***	1.9 ***	1.8 ***
All	3.6 ***	2.9 ***	3.0 ***	3.6 ***

Table S4. Correlation coefficients between the percentage of people of color and LST by city over time (*p-value <0.05, **p-value<0.01, ***p-value<0.001).

City	1990	2010	2020
Bridgeport	0.39 ***	0.36 ***	0.39 ***
Danbury	0.34 ***	0.47 ***	0.56 ***
Hamden	0.28 ***	0.48 ***	0.47 ***
Hartford	-0.06 -	-0.07 -	-0.07 -
Manchester	0.22 ***	0.46 ***	0.45 ***
New Britain	0.25 ***	0.34 ***	0.39 ***
New Haven	0.38 ***	0.23 ***	0.22 ***
Norwalk	0.55 ***	0.59 ***	0.60 ***
Stamford	0.52 ***	0.64 ***	0.65 ***
Waterbury	0.41 ***	0.33 ***	0.33 ***
All	0.44 ***	0.48 ***	0.51 ***

Table S5 Disparities in air conditioner (AC) rate and tree cover between people of color communities and white communities over time (*p-value <0.05, **p-value<0.01, ***p-value<0.001).

City	AC rate	Tree cover	Vegetation cover
Bridgeport	16% ***	13% ***	13% ***
Danbury	16% ***	20% ***	19% ***
Hamden	17% ***	18% ***	16% ***
Hartford	20% ***	4% **	0% -
Manchester	4% *	10% ***	10% ***
New Britain	5% **	6% ***	11% ***

New Haven	20% ***	2% -	3% *
Norwalk	29% ***	14% ***	14% ***
Stamford	26% ***	22% ***	21% ***
Waterbury	15% ***	11% ***	11% ***
All	22% ***	15% ***	15% ***

Table S6. Correlation coefficients between the percentage of people of color and AC rate, tree cover, and vegetation cover, respectively (*p-value <0.05, **p-value<0.01, ***p-value<0.001).

City	AC rate	Tree cover	Vegetation cover
Bridgeport	-0.37 ***	-0.39 ***	-0.38 ***
Danbury	-0.30 ***	-0.55 ***	-0.54 ***
Hamden	-0.34 ***	-0.52 ***	-0.49 ***
Hartford	-0.42 ***	0.04 -	0.13 ***
Manchester	-0.06 -	-0.44 ***	-0.43 ***
New Britain	-0.12 **	-0.35 ***	-0.41 ***
New Haven	-0.39 ***	-0.07 *	-0.12 ***
Norwalk	-0.58 ***	-0.35 ***	-0.40 ***
Stamford	-0.56 ***	-0.62 ***	-0.62 ***
Waterbury	-0.42 ***	-0.41 ***	-0.41 ***
All	-0.47 ***	-0.47 ***	-0.46 ***

Table S7. Disparities in NDVI between people of color communities and white communities over time (*p-value <0.05, **p-value<0.01, ***p-value<0.001).

City	NDVI mean	NDVI 1990	NDVI 2010	NDVI 2020
Bridgeport	0.11 ***	0.09 ***	0.11 ***	0.12 ***
Danbury	0.13 ***	0.19 ***	0.10 ***	0.14 ***
Hamden	0.12 ***	0.11 ***	0.11 ***	0.12 ***
Hartford	0.02 -	-0.01 -	0.03 *	0.01 -
Manchester	0.07 ***	0.18 -	0.08 ***	0.07 ***
New Britain	0.08 ***	0.06 **	0.06 ***	0.08 ***
New Haven	0.02 **	0.08 ***	0.02 *	0.02 *
Norwalk	0.14 ***	0.16 ***	0.16 ***	0.15 ***
Stamford	0.21 ***	0.19 ***	0.21 ***	0.23 ***
Waterbury	0.08 ***	0.10 ***	0.08 ***	0.08 ***
All	0.14 ***	0.12 ***	0.12 ***	0.14 ***

Table S8. Correlation coefficients between the percentage of people of color and NDVI by city over time (*p-value <0.05, **p-value<0.01, ***p-value<0.001).

City	1990	2010	2020

Bridgeport	-0.42 ***	-0.38 ***	-0.38 ***
Danbury	-0.34 ***	-0.43 ***	-0.55 ***
Hamden	-0.31 ***	-0.48 ***	-0.49 ***
Hartford	0.08 *	0.05 -	0.11 **
Manchester	-0.24 ***	-0.45 ***	-0.45 ***
New Britain	-0.23 ***	-0.34 ***	-0.39 ***
New Haven	-0.34 ***	-0.08 **	-0.09 **
Norwalk	-0.54 ***	-0.53 ***	-0.49 ***
Stamford	-0.55 ***	-0.65 ***	-0.64 ***
Waterbury	-0.37 ***	-0.36 ***	-0.37 ***
All	-0.41 ***	-0.46 ***	-0.50 ***

Table S9. Correlation coefficients between LST mean and NDVI mean, and LST change and NDVI change (*p-value <0.05, **p-value<0.01, ***p-value<0.001).

City	LST mean VS NDVI mean	LST change VS NDVI change
Bridgeport	-0.79 ***	-0.25 ***
Danbury	-0.87 ***	-0.54 ***
Hamden	-0.96 ***	-0.53 ***
Hartford	-0.77 ***	-0.37 ***
Manchester	-0.93 ***	-0.63 ***
New Britain	-0.93 ***	-0.40 ***
New Haven	-0.78 ***	-0.33 ***
Norwalk	-0.79 ***	-0.28 ***
Stamford	-0.92 ***	-0.48 ***
Waterbury	-0.93 ***	-0.45 ***
All	-0.87 ***	-0.36 ***

Table S10. Tree cover, vegetation (Veg) cover, and accuracies of high-resolution tree cover maps and vegetation cover maps

City	Tree cover	Tree producer's accuracy	Tree user's accuracy	Tree overall accuracy	Veg cover	Veg producer's accuracy	Veg user's accuracy	Veg overall accuracy
New Haven	30.3%	84%	84%	89%	47.8%	94%	94%	94%
Danbury	57.0%	96%	87%	92%	67.4%	99%	90%	94%
Hamden	59.6%	93%	90%	90%	75.0%	99%	95%	96%
Norwalk	42.6%	89%	80%	88%	55.4%	96%	87%	91%
Waterbury	50.7%	95%	89%	92%	60.9%	97%	93%	94%
Stamford	45.8%	93%	89%	91%	54.2%	94%	94%	96%
Hartford	19.4%	82%	86%	93%	42.2%	95%	85%	92%
Manchester	43.1%	82%	93%	88%	63.7%	99%	97%	97%

New Britain	32.7%	82%	81%	87%	53.6%	96%	99%	97%
Bridgeport	25.5%	90%	83%	93%	36.7%	91%	95%	94%

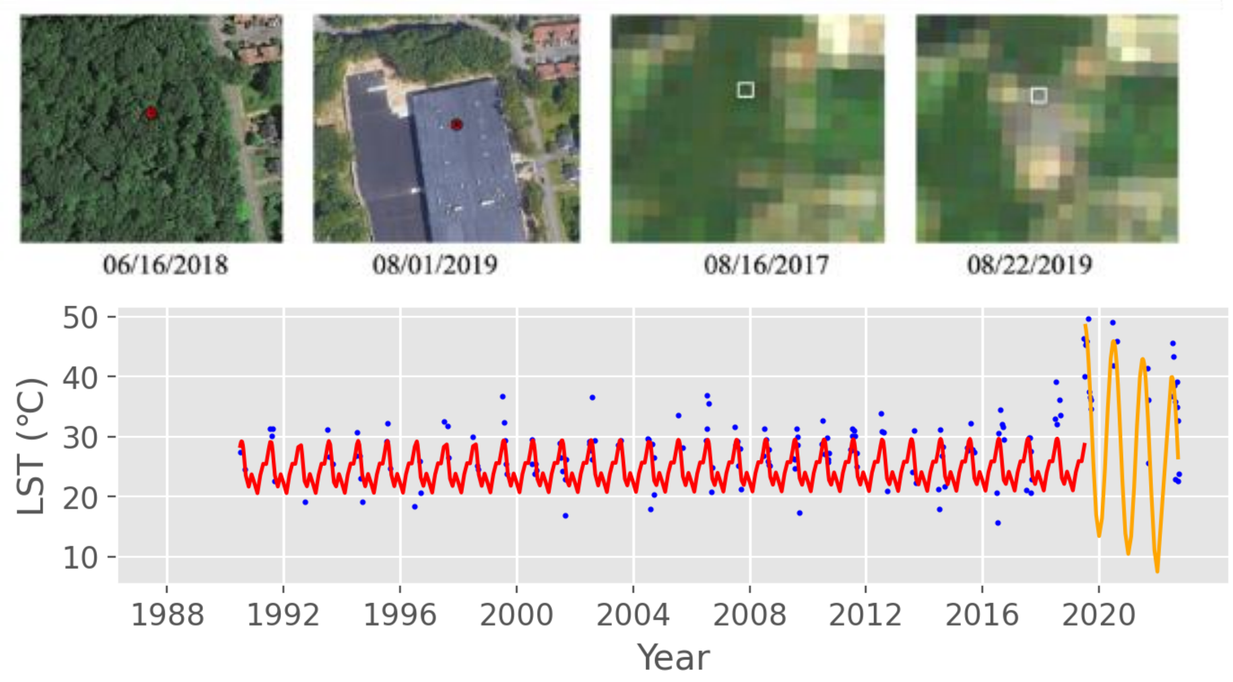

Figure S2. LST increases due to tree loss and the construction of a building. The first two snapshots are high-resolution images before and after the change. In these two images, the red points show the center of this pixel. The last two snapshots are Landsat imagery before and after the change. The white squares show the pixel location. In the time series plot, blue points are Landsat observations of this location. Red and orange curves are CCDC model fitting curves.

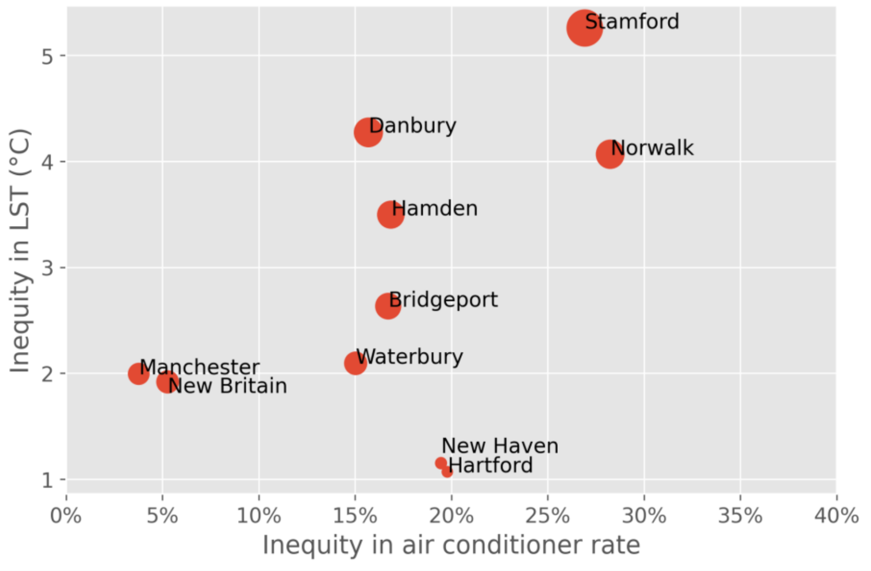

Figure S3. Disparities between people of color communities and white communities in different cities. The sizes of the markers are proportional to NDVI.

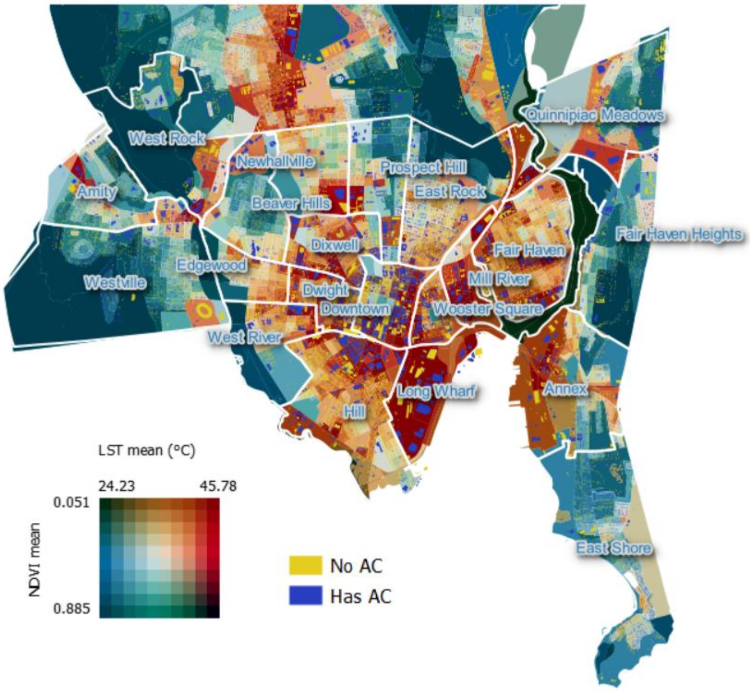

(a) New Haven

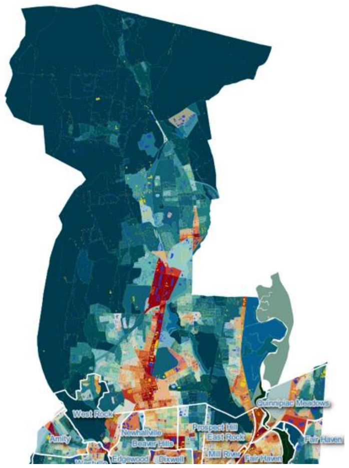

(b) Hamden

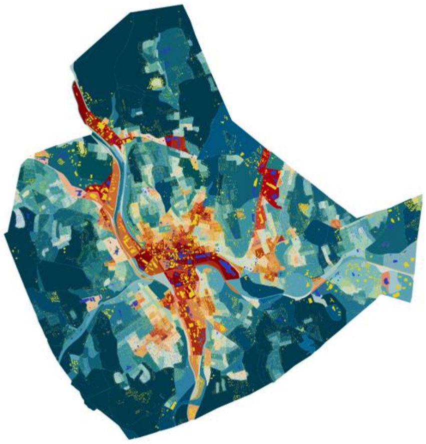

(c) Waterbury

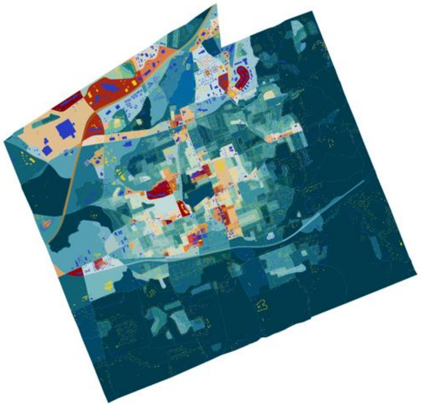

(d) Manchester

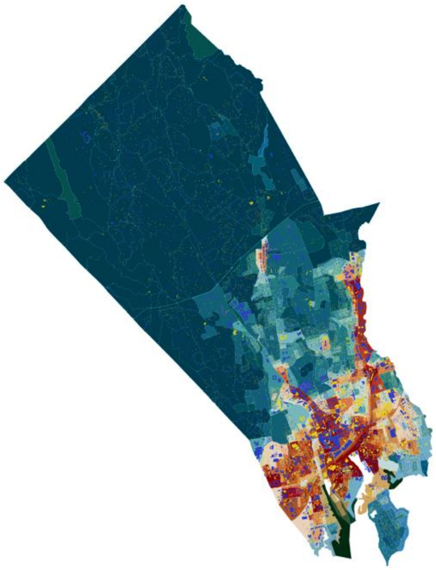

(e) Stamford

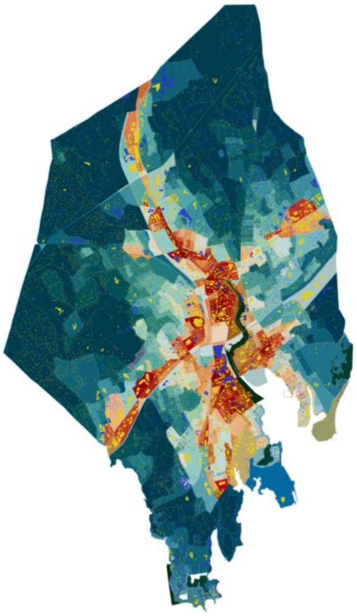

(f) Norwalk

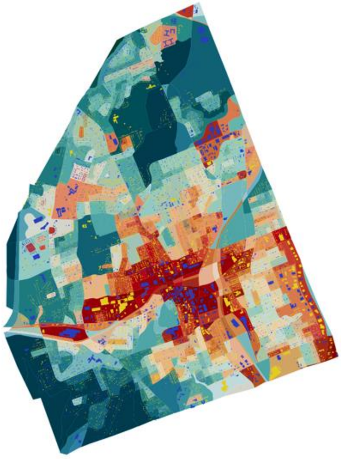

(g) New Britain

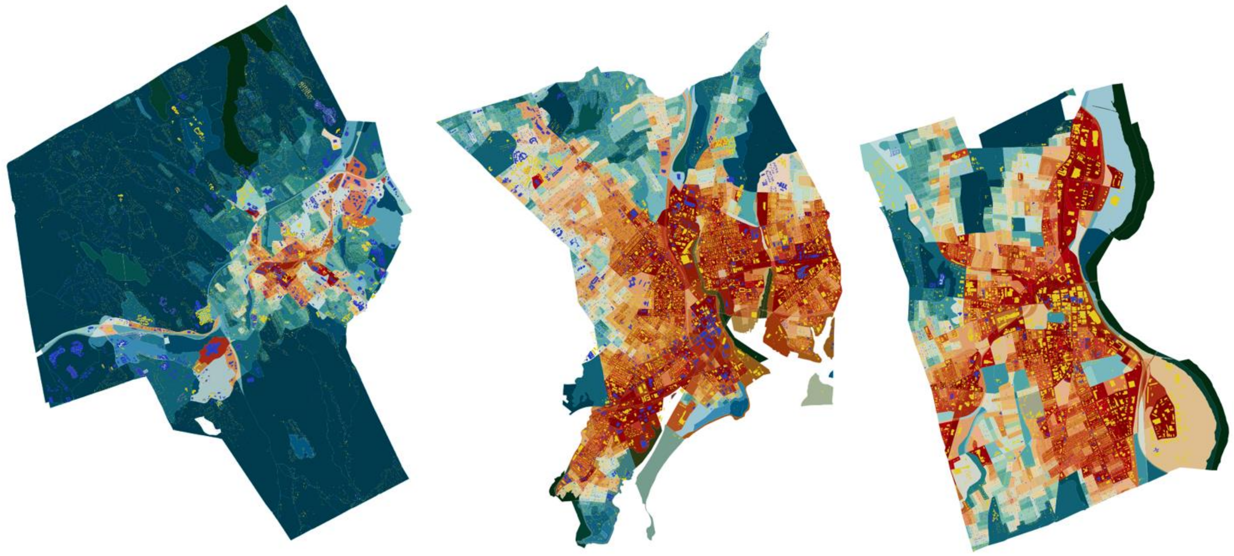

(h) Danbury (i) Bridgeport (j) Hartford

Figure S4. Map of LST, NDVI, and AC. The legend is shown in Figure S3. (a).
